# Low Tongue Posture Improvement Effect of Orofacial Myofunctional Therapy Comprehensive Study of Nasal Ventilation Condition Using Computational Fluid Dynamics and Dental Arch Morphology

**DOI:** 10.1111/ocr.70000

**Published:** 2025-07-01

**Authors:** Kei Maeo, Takamasa Kitamura, Wakana Kohira, Yukari Suzuki, Yoshihito Yamakawa, Kimiko Ueda, Hitomi Ishii, Ryuzo Kanomi, Tomonori Iwasaki

**Affiliations:** ^1^ Department of Pediatric Dentistry, Institute of Biomedical Sciences Tokushima University Graduate School Tokushima Japan; ^2^ Kanomi Orthodontic Office Himeji Japan

**Keywords:** computational fluid dynamics, low tongue posture, myofunctional therapy, nasal airway obstruction, rapid maxillary expansion

## Abstract

**Objectives:**

Oral myofunctional therapy (OMFT) has been proposed as a potential treatment for improving low tongue posture (LTP) and alleviating obstructive sleep apnea. However, its effectiveness remains uncertain. This study aimed to quantify the effects of OMFT on LTP.

**Materials and Methods:**

This study analysed pre‐ and post‐treatment cone‐beam computed tomography images from 43 children with LTP treated with rapid maxillary expansion (RME) only (mean age: 9.09 years, no‐OMFT group), 46 children with LTP treated with RME plus OMFT (mean age: 9.40 years, OMFT group), and 20 children (mean age: 9.87 years, control group). The primary outcomes measured were intraoral airway volume (an indicator of LTP), nasal airway pressure differences (assessed using computational fluid dynamics), and maxillary dental arch width. Comparisons were made among groups, with the frequency and relationship between these measures investigated.

**Results:**

There were no significant differences between OMFT and no‐OMFT groups before treatment. Post‐treatment, the OMFT group showed significantly smaller intraoral airway volume (0.66 cm^3^) compared to the no‐OMFT group (1.34 cm^3^). Nasal airway pressure drop was also significantly lower in the OMFT group (44.2 Pa) than in the no‐OMFT group (143.3 Pa).

The LTP improvement rate was significantly higher in the OMFT group (76.1%) than in the no‐OMFT group (51.2%). Similarly, the improvement rate of nasal airway obstruction was significantly higher in the OMFT group than in the no‐OMFT group.

**Conclusion:**

OMFT improves LTP and nasal airway obstruction. Improvements in nasal airway obstruction by OMFT are likely correlated with the observed improvements in LTP.

## 1 Introduction

Adenoid tonsillectomy is the first‐line treatment modality for paediatric obstructive sleep apnea (OSA) [1]. However, some paediatric patients do not benefit from this surgery, with the patients continuing to breathe through the mouth [2] not only because of nasal obstruction, but also because of weak oral muscles that cause the mouth to remain open during the day [3]. Therefore, an important treatment aspect is determining whether the cause of mouth breathing is nasal obstruction or muscle function such as weak oral muscles. For the former cause, nasal treatment is required, whereas for the latter cause, orofacial myofunctional therapy (OMFT) is proposed [4]. OMFT has been reported to improve the symptoms of children diagnosed with OSA based on clinical symptoms and polysomnography by improving the muscle function of the tongue and perioral muscles [5, 6, 7, 8]. These benefits are mainly exerted through improvement of nasal breathing and low tongue posture (LTP) [9, 10, 11]. Tongue position has been reported to be closely related to mouth breathing [4, 12]. Furthermore, in the context of the relationship between nasal ventilation and tongue posture, when nasal breathing disorders do not improve after rapid maxillary expansion (RME), the LTP also does not improve. Conversely, when nasal breathing improves, the LTP improves [13]. This suggests that nasal breathing disorders are closely related to tongue posture [13].

Meanwhile, in children without breathing disorders, the narrowing of the maxillary dental arch is related to an LTP [14]. The LTP is corrected to a high tongue posture after the maxillary dental arch is expanded by RME. As such, the position of the tongue is considered to be related to mouth breathing [4, 12], nasal obstruction [13] and narrowing of the maxillary dental arch [14]. However, the mechanisms through which OMFT improves nasal obstruction and LTP have not yet been fully clarified. Therefore, comprehensive research that incorporates a three‐dimensional quantitative evaluation of tongue posture, nasal ventilation and maxillary dental arch width is needed to gain a deeper understanding of the effects of OMFT on nasal obstruction and LTP. Nasal septal deviation [15] and nasal mucosal hypertrophy [16] also affect nasal ventilation, and thus, these factors also need to be considered.

The present study aimed to clarify the effects of OMFT on nasal obstruction and LTP. Towards this goal, we conducted a comprehensive three‐dimensional evaluation of tongue posture, nasal airflow (as evaluated using computational fluid dynamics [CFD]), and maxillary dental arch width in children who only underwent RME, those who underwent both RME and OMFT, and normal children.

## 2 Material and Methods

### 2.1 Ethical Approval and Study Design

This retrospective study was approved by the Institutional Review Board of Tokushima University (4393‐4) and was conducted according to the tenets of the Declaration of Helsinki. The requirement for informed consent was waived owing to the retrospective nature of the study.

### 2.2 Participant and Control Selection

Participants were retrospectively selected from the archives of the orthodontic clinic at the collaborating institutions. Cone‐beam computed tomography (CBCT) images were obtained at two time points: T1 (before RME) and T2 (after RME or RME + OMFT). The inclusion criteria were as follows: (1) age 7–11 years at T1 and 10–14 years at T2; (2) LTP [13] (intraoral airway volume [IAv, i.e., airway volume between the palate and tongue] > 1 cm^3^) at the time of initial CBCT imaging; (3) narrow maxillary dentition requiring maxillary expansion of approximately 5 mm as part of orthodontic treatment (passive retention before the full orthodontic treatment device was not used); (4) no prior orthodontic treatment; and (5) normal development without systemic diseases or craniofacial syndromes. The exclusion criteria were as follows: (1) a history of adenoid hypertrophy [17] or palatine tonsil hypertrophy [18]; (2) a history of adenoidectomy or tonsillectomy; (3) the presence of systemic disease [15]; (4) craniofacial anomalies [15]; (5) temporomandibular joint disorders [19]; and (6) CBCT imaging revealing abnormalities (e.g., tongue positions other than swallowing or other resting positions) [19].

The control group involved children with normal tongue posture who had not used orthodontic appliances (e.g., quad helix), functional orthodontic appliances, or rapid expansion appliances by T2. CBCT scans for the control group were performed at ages corresponding to T1 and T2, and the control group was selected so that the distribution of the OMFT and no OMFT groups with respect to sex and maxillofacial dentition occlusion would be similar. The exclusion criteria were as follows: (1) deformities of the uvula or other craniofacial deformities and craniofacial or growth abnormalities, (2) systemic diseases and (3) temporomandibular disorders.

From the 558 children who received their first examination, 89 children diagnosed with LTP were included in the current study. They were divided into two groups as the no OMFT group (i.e., those who received only RME, *n* = 43 boys) and the OMFT group (i.e., those who received RME plus OMFT, *n* = 46 boys). In addition, 20 children (20 boys) were selected as the control group. Thus, a total of 109 children were included in the current study. Before 2020, OMFT was only performed when deemed particularly necessary. However, after 2020, OMFT is commonly performed after RME based on recent evidence supporting its effectiveness for paediatric OSA [5, 6, 7, 8]. Given these differences, the effect of OMFT could be compared by comparing the cohort before and after 2020. The groups were comparable with respect to sex, age and dentition. Other than the active introduction of OMFT, there were no significant changes in RME or other treatment methods during the study period (2013–2024).

### 2.3 OMFT Training Protocol

OMFT was performed to increase lip tension and optimise tongue position [8]. To improve lip closure strength, the child was asked to hold a button with a diameter of approximately 3 cm between their lips and teeth. The button was then pulled horizontally with the maximum force that did not cause it to pop out of the child's mouth for 10 s. The pulling was weakened and repeated 20 times, with the child instructed to do this twice each morning and evening. This lip training was carried out from the time the RME was fitted. The tongue exercises were started after the RME was removed. The exercises included tongue spots, tongue clicks, tongue lateral movements, tongue movements using a spoon, tongue twisters, tongue push‐ups and tongue exercises. These two exercises for the lips and tongue took 20 min per day and were carried out for approximately 6 months [10].

### 2.4 CBCT Scan

All CBCT scans were performed with the participants seated, ensuring that the Frankfort horizontal plane was parallel to the floor [20]. CBCT was indicated for its ability to minimise radiation exposure and provide a comprehensive 3D evaluation. Scans were performed pre‐RME to examine maxillofacial morphology, nasal and pharyngeal airways, sinus conditions and dental problems. After RME, immediately before proceeding to the second phase of orthodontic treatment, CBCT was repeated to reassess 3D maxillofacial morphology, airways and dental conditions, with particular focus on root resorption [21] and buccal alveolar bone loss [22, 23] associated with RME. Participants with swallowing or other tongue movements based on the CBCT images obtained were additionally excluded.

### 2.5 Morphological Evaluations

**FIGURE 1:**
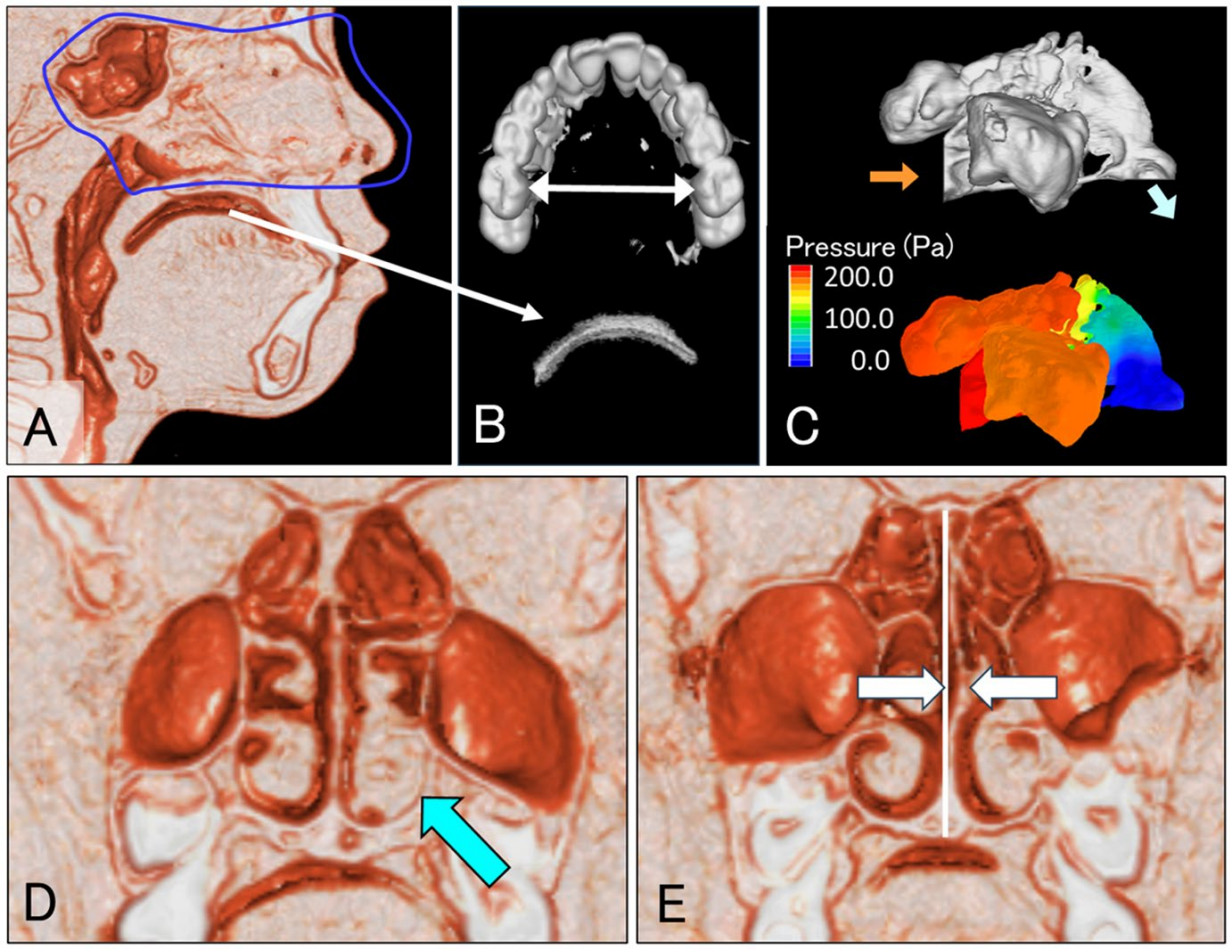
Measurement of the maxillary dental arch width, low tongue posture and nasal ventilation using computational fluid dynamics, nasal mucosa hypertrophy and nasal septum. (A) Measurement sites for nasal and oral airway volume. (B) Upper: Measurement of the width between the maxillary first molars. Lower: Oral airway viewed from the right side. (C) Upper: 3D model of nasal airway. Lower: Analysis results of nasal airway using computational fluid dynamics. (D) Nasal mucosa hypertrophy. (E) Measurement of nasal septum deviation.

The maxillary dental arch width (intramaxillary molar width) and the intraoral airway volume (IAv), which represents the airway space between the tongue and palate, were measured using CBCT (Figure 1) [13]. Previous studies have shown that an IAv of ≥ 1 cm^3^ is associated with malocclusions and nasal airway obstruction [13, 20, 24, 25], whereas an IAv < 1 cm^3^ is seen in cases without such issues or with improved nasal airflow. Therefore, in this study, we defined LTP as an IAv ≥ 1 cm^3^ in a morphological and functional sense. After treatment, LTP improvement was defined as an IAv < 1 cm^3^. Additionally, nasal mucosa hypertrophy and nasal septum deviation were measured (Figure 1) [16].

### 2.6 Nasal Ventilation Condition

Nasal ventilation conditions were measured using the nasal airway CFD model. The constructed 3D nasal airway model was simulated to estimate the nasal pressure drop corresponding to an exhalation rate of 200 mL/s (Figure 1) [26, 27, 28].

### 2.7 Definition of Nasal Airway Obstruction

Previous studies [29, 30] have reported a nasal airway resistance of 0.5 Pa/mL/s in elementary school children with nasal airway obstruction. Therefore, in our flow setting (200 mL/s), nasal airway obstruction was defined as a pressure drop ≥ 100 Pa. Furthermore, when the continuity of both nostrils in the 3D nasal airway model was blocked, it was identified as nasal airway obstruction (3D obstruction). After treatment, nasal airway obstruction improvement was defined as a pressure drop < 100 Pa. To evaluate the measurement errors in maxillary dental arch width, IAv and nasal pressure, the first evaluator (K.M.) verified the measurement accuracy with another author (T.I.) for 10 CT images randomly selected from 89 participants. The same operator (K.M.) then measured each item twice within a week. After these repeated measurements, all measurement errors were determined using Dahlberg's formula [31]. The measurement errors of the images obtained in this study were 0.41 mm, 0.223 mm^3^ and 0.534 Pa for maxillary dental arch width, oral airway volume and maximal nasal airway pressure drop, respectively. All repeated analyses indicated that the measurement errors were negligible.

### 2.8 Statistics

Descriptive statistics were calculated at the group level for age, maxillary dental arch width, IAv and nasal pressure at T1, T2 and T2‐T1. The normality of data distribution was confirmed using the Shapiro–Wilk test. Analysis of variance (ANOVA) was used to compare age, maxillary dental arch width and IAv at T1, T2 and T2‐T1 among the OMFT, no OMFT and control groups. The Kruskal‐Wallis test was used for similar analysis of nasal pressure. For each group, changes in age, maxillary dental arch width and IAv from T1 to T2 were evaluated using a paired t‐test, while changes in nasal airway pressure from T1 to T2 were evaluated using a Wilcoxon signed‐rank test. The relationship between IAv and maxillary dental arch width was evaluated using the Pearson correlation coefficient, and the relationship between IAv and nasal airway ventilation was evaluated using the Spearman correlation coefficient. Furthermore, (1) the rate of improvement of LTP and nasal airway obstruction in the OMFT group and no OMFT group and (2) the rate of improvement of nasal airway obstruction and LTP in the OMFT group and no OMFT group in participants with nasal mucosal thickening and nasal septal deviation were analysed using the chi‐square test.

Additionally, to clarify the relationship between posttreatment nasal obstruction and LTP, we also analysed participant distribution according to the presence or absence of nasal obstruction (nasal airway pressure 100 Pa) and LTP (IAv 1 cm^3^), using the chi‐square test. In addition, to evaluate the relationship among these four conditions and dental arch width, ANOVA was used to compare the width of the dental arches among the four groups. All multiple comparisons were performed using Bonferroni correction. Based on the hypothesis that RME alone or in combination with OMFT improved obstruction of the hypopharynx and nasal airway, the difference in treatment‐related changes in nasal airway pressure (decrease in nasal airway pressure) was set as the basis for calculating the sample size. Power analysis (1‐β error = 0.80, *α* = 0.05, two‐tailed test) was used to calculate the required number of participants. The appropriate sample size for each group was determined to be 18 participants, for a total of 54 participants. All statistical analyses were performed using IBM SPSS Statistics (version 28.0.1.0, IBM, Armonk, NY). *p* < 0.05 was considered significant.

## 3 Results

### 3.1 Participant Characteristics

Age, sex and maxillofacial morphology (Class I, II and III) were not significantly different among the three groups (Table 1). The maxillary dentition width diameter at T1 was not significantly different between the OMFT and no OMFT groups, but it was significantly smaller in both groups than in the control group (Table 1). However, the diameter at T2 was not significantly different among the three groups. At T2–T1, there was no significant difference between the OMFT and no OMFT groups, but the values were significantly larger in both groups I than in the control group. With respect to the change from T1 to T2, the maxillary first molar width diameter was not significantly changed in the control group, but it was significantly increased in the OMFT and no OMFT groups. For IAv at T1, there was no significant difference between the OMFT and no OMFT groups, but the values were significantly larger in both groups (2.22 and 2.09 cm^3^, respectively) than in the control group (0.00 cm^3^) (Table 1). The IAv at T2 was significantly different among the three groups, with the control group having the smallest IAv (0.01 cm^3^), followed by the OMFT group (0.66 cm^3^). The IAv was the largest in the no OMFT group (1.34 cm^3^). The IAv at T2‐T1 was significantly reduced in the OMFT group, with the reduction being greater not only compared with the control group but also with the no OMFT group.

**TABLE 1:** Comparison between the OMFT and no OMFT groups.

		Control (*n* = 20)		MFT (*n* = 46)		No‐MFT (*n* = 43)		ANOVA	Post hoc
			T1 vs. T2^a^		T1 vs. T2^a^		T1 vs. T2^a^	*F* value *p*	Bonferroni
Age (year)									
T1	Mean	9.87		9.43		9.09		2.526	
	SD	1.74		1.29		1.07		0.085	
T2	Mean	12.13		11.75		11.80		0.622	
	SD	2.18		1.02		0.99		0.539	
T2–T1	Mean	2.26	*t* = −15.321	2.33	*t* = −18.904	2.71	*t* = −19.399	3.032	
	SD	0.66	*p* < 0.001	0.84	*p* < 0.001	0.92	*p* < 0.001	0.052	
Maxillary first molar width (mm)									
T1	Mean	35.18		33.21		33.39		8.123	12, 13
	SD	1.55		2.83		2.71		< 0.001	
T2	Mean	36.43		36.24		36.36		0.039	
	SD	1.58		2.80		3.13		0.962	
T2–T1	Mean	0.54	*t* = −2.317	3.02	*t* = −13.109	2.96	*t* = −13.535	24.007	12, 13
	SD	1.04	*p* < 0.001	1.56	*p* < 0.001	1.43	*p* < 0.001	< 0.001	
Intraoral airway volume (cm^3^)									
T1	Mean	0.00		2.22		2.09		56.029	12, 13
	SD	0.00		1.04		0.75			
T2	Mean	0.00		0.66		1.34		< 0.001	12, 13, 23
	SD	0.00		1.11		1.55		< 0.001	
T2–T1	Mean	0.00	—	−1.56	*t* = 7.526	−0.75	*t* = 3.151	6.628	12, 23
	SD	0.00		1.41	*p* < 0.001	1.57	*p* = 0.003	0.012	

			T1 vs. T2^b^		T1 vs. T2^b^		T1 vs. T2^b^	Kruskal‐Wallis‐test *H* value *p*	Post hoc Bonferroni
Nasal airway pressure (Pa)									
T1	Mean	45.6		298.3^d^		493.7^c^		5.205	13
	SD	34.5		430.9		684.5		< 0.001	
T2	Mean	40.1		44.2^c^		143.3^c^		6.768	13, 23
	SD	39.0		37.4		214.6		0.017	
T2–T1	Mean	7.3^e^	*Z* = 0.299	259.5^e^	*Z* = −4.535	350.3^c^	*Z* = −3.895	8.080	13
	SD	47.5	*p* = 0.763	437.9	*p* < 0.001	596.3	*p* < 0.001	< 0.001	

*Note:* 12: control group versus OMFT group, 13: control group versus no OMFT group, 23: MFT group versus no OMFT group.

^a^ T1 versus T2 paired t‐test *p* < 0.01.

^b^ T1 versus T2 Wilcoxon tank test *p* < 0.001.

^c^ One participants diagnosed with 3D obstruction are excluded from the analysis.

^d^ Six participant diagnosed with 3D obstruction is excluded from the analysis.

^e^ Seven participants diagnosed with 3D obstruction are excluded from the analysis.

Regarding the change in IAv from T1 to T2, it was not significantly changed in the control group, but the values were significantly decreased in the OMFT (−1.56 cm^3^) and no OMFT (−0.75 cm^3^) groups. The nasal airway pressure at T1 was 493.8 Pa in the no OMFT group, significantly higher than the 100 Pa threshold for nasal obstruction. This value was also significantly higher than that in the control group (45.6 Pa). However, the values were not significantly different between the no OMFT and OMFT groups (Table 1). At T2, the nasal airway pressure was significantly higher in the no OMFT group (143.3 Pa) than in the control (40.1 Pa) and OMFT (44.2 Pa) groups. At T2–T1, the value was significantly lower in the no OMFT group than in the control group. There was no significant change in nasal pressure from T1 to T2 in the control group, but it was significantly decreased in the OMFT and no OMFT groups.

### 3.2 Improvement Rate of Low Tongue Posture and Nasal Obstruction With and Without OMFT

The OMFT group showed significantly higher rates of improvement in LTP (76.1%) and nasal airway obstruction (81.3%) compared to the no OMFT group (51.2% and 48.4%, respectively) (Table 2). Similar results were observed in the participants with nasal mucosa hypertrophy and nasal septal deviation.

**TABLE 2:** Improvement rates for low tongue posture and nasal airway obstruction in the OMFT and no OMFT groups.

			MFT	No‐MFT	Chi‐square test *p*
All case (89)	Low tongue	Improve (*n*)	35	22	*χ* ^2^(1) = 5.996 *p* = 0.014
		Not improve (*n*)	11	21	
		Improvement incidence	76.1%	51.2%	
	Nasal obstruction^a^	Improve (*n*)	26	15	*χ* ^2^(2) = 7.558 *p* = 0.023
		Not improve (*n*)	6	16	
		No obstruction^b^ (*n*)	14	12	
		Improvement incidence	81.3% (26/32)	48.4% (15/31)	
Nasal mucosa hypertrophy case (45)	Low tongue	Improve (*n*)	18	5	*χ* ^2^(1) = 8.091 *p* = 0.004
		Not improve (*n*)	8	14	
		Improvement incidence	69.2%	26.3%	
	Nasal obstruction^a^	Improve (*n*)	14	4	*χ* ^2^(2) = 13.186 *p* = 0.001
		Not improve (*n*)	3	12	
		No obstruction^b^ (*n*)	9	3	
		Improvement incidence	82.4% (14/17)	25% (4/16)	
Nasal septum deviation case (23)	Low tongue	Improve (*n*)	10	2	*χ* ^2^(1) = 7.340 *p* = 0.012
		Not improve (*n*)	3	8	
		Improvement incidence	76.9%	20.0%	
	Nasal obstruction^a^	Improve (*n*)	8	0	*χ* ^2^(2) = 9.920 *p* = 0.007
		Not improve (*n*)	2	6	
		No obstruction^b^ (*n*)	3	4	
		Improvement incidence	80% (8/10)	0% (0/6)	

^a^ Children with nasal airway obstruction before treatment.

^b^ No obstruction; T1 and T2 no nasal obstruction.

### 3.3 Relationship of IAv With Nasal Ventilation and Maxillary Dental Arch Width

**FIGURE 2:**
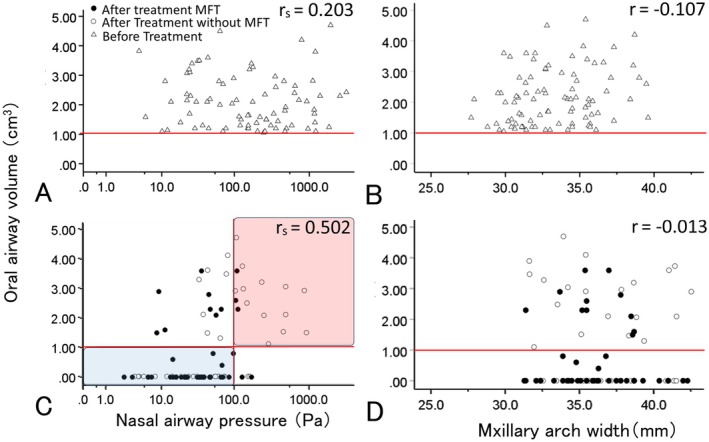
Relationship among intra‐oral airway volume (IAv), nasal airway pressure and arch width before and after treatment. (A) There is a significant correlation between the IAv and nasal airway pressure before treatment, but the relationship is weak (rs = 0.203). (B) There is no significant correlation between the IAv and dental arch width before treatment (rs = −0.107). (C) There is a significant positive correlation between the IAv and nasal airway pressure after treatment (rs = 0.502). The nasal airway pressure of 53 participants with an IAv of < 1 cm^3^ (an indicator of a low tongue posture) is < 100 Pa, and only five participants show a pressure of ≥ 100 Pa. This suggests that a low tongue posture is not the cause of nasal airway obstruction (blue range). Meanwhile, among the participants with nasal airway obstruction (≥ 100 Pa), 17 participants had an IAv of ≥ 1 cm^3^, and four participants had an IAv of < 1 cm^3^, with a significant difference between the two groups. (D) There is no significant correlation between posttreatment IAv and dental arch width, regardless of whether or not MFT is used (rs = −0.013).

Before treatment, there was a weak significant correlation (rs = 0.203) between IAv and nasal airway pressure (Figure 2A) and no significant correlation between IAv and maxillary dental arch width (*r* = −0.107) (Figure 2B). After treatment, IAv was significantly correlated with nasal airway pressure (rs = 0.502, Figure 2C) but not with maxillary dental arch width (*r* = −0.013, Figure 2D).

### 3.4 Relationship Among Nasal Obstruction, Tongue Position and Dental Arch Width After Treatment

Among participants with an IAv ≤ 1 cm^3^, many participants had an IAv of ≤ 100 Pa; meanwhile, among participants with an IAv ≥ 1 cm^3^, many participants had an IAv of ≤ 100 Pa, with a significant difference in distribution (Figure 2C, Table 3). Maxillary dental arch width was not significantly different among the four groups (Table 3).

**TABLE 3:** Relationship between nasal airway obstruction and low tongue posture after treatment and comparison of maxillary dental arch width.

		Low tongue posture						
		None			Present			Chi‐square test *p*
		Case (*n*)	Maxillary dental arch width (mm)		Case (*n*)	Maxillary dental arch width (mm)		
			Mean	SD		Mean	SD	
Nasal airway obstruction	None	52	36.14	2.93	15	36.32	2.96	*χ* ^2^(1) = 21.655 < 0.001
	Present	5	36.70	2.63	17	36.62	3.29	

*Note:* Maxillary dental arch width: the width between the maxillary first molars, No significant difference in maxillary first molar width among the four groups (*F* = 0.140, *p* = 0.936).

### 3.5 Relationship Between Treatment‐Related Improvement of Nasal Airway Obstruction and of Low Tongue Posture

Of the 41 participants in whom nasal airway obstruction improved, 34 participants showed improvement in tongue posture. Meanwhile, among the 22 participants in whom nasal airway obstruction did not improve, five participants showed improvement in tongue posture. The distribution of participants with and without improvement in nasal airway obstruction and tongue posture was significantly different (data not shown).

## 4 Discussion

### 4.1 Main Findings

**FIGURE 3:**
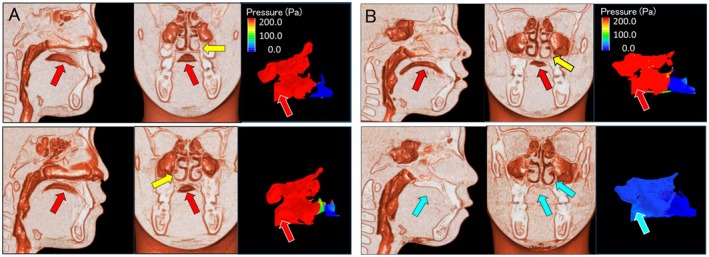
Differences in tongue position and nasal airflow according to treatment method. Sagittal section, left anterior view, nasal airflow. (A) In this participant, nasal obstruction and low tongue posture are not improved by the rapid expansion device alone. Upper row: Before treatment in a participant in whom only a rapid maxillary expander is used. The low tongue posture (red arrow), thickened nasal mucosa (yellow arrow), and high nasal pressure (red arrow) indicate nasal obstruction. Lower row: After treatment in a participant in whom only a rapid maxillary expander is used. The maxillary dental arch is expanded laterally, but nasal airway obstruction does not improve (red arrow), the tongue position remains low (red arrow), and mild nasal mucosal hypertrophy (yellow arrow) is observed. (B) Participant with nasal airway obstruction and low tongue posture improved by maxillary rapid expansion device and oral myofunctional therapy. Upper: Before treatment in a participant in whom maxillary rapid expansion device and oral myofunctional therapy are used in combination. The tongue position is low (red arrow), and nasal mucosal hypertrophy (yellow arrow) was observed. Nasal secretion analysis show high pressure (red arrow), indicating nasal airway obstruction. Lower: After treatment using a rapid expansion device and oral muscle function therapy, the maxillary dental arch expands laterally to the same extent as with treatment using a rapid expansion device alone, but nasal airway obstruction improves (blue arrow), tongue posture improves (blue arrow), and nasal mucosal hypertrophy disappears (blue arrow).

Previous studies have reported that RME improves nasal obstruction and LTP [13, 24]. However, the present study found that compared to RME alone, the combination of OMFT and RME was associated with a greater reduction in nasopharyngeal pressure and IAv and greater improvement in nasal obstruction and LTP (Figures 2 and 3, Tables 1 and 2). The relationship between nasal airway pressure posttreatment and IAv shows that the thresholds for nasal airway obstruction and LTP were 100 Pa and 1 cm^3^, respectively (Figure 2C). The 100 Pa indicated by CFD was shown to have a flow rate of 200 mL/s, and thus, the resistance value corresponds to 0.5 Pa/mL/s, consistent with the nasal airway obstruction values reported previously [29, 30]. Therefore, the results of the CFD analysis used in the present study are not clinical data, but the clinical efficacy of the results is confirmed based on previous findings [28, 32, 33]. However, some participants with IAv < 1 cm^3^ had nasal airway pressure of ≥ 100 Pa, although these participants had relatively low nasal airway pressure. This also shows that an IAv of < 1 cm^3^ is a valid diagnostic indicator of nasal airway obstruction (Figure 2C). This study highlights a close relationship between the improvement of nasal airway obstruction and LTP posttreatment, suggesting that OMFT improves nasal airway obstruction and, by extension, LTP. In some participants, tongue position did not improve with OMFT (Figure 2C,D, Table 2). The possible reasons for this include refractory nasal obstruction; the degree of compliance with OMFT; the persistence of LTP despite the absence of nasal airway obstruction (habitual mouth breathing not due to nasal airway obstruction) [34]; and the influence of soft tissues such as the tongue, lip muscles and the lingual frenulum. Future studies should consider patients who do not show such improvements not only on CBCT imaging but also on detailed clinical examinations.

### 4.2 Regarding the Relationship Between LTP and Maxillary Dental Arch Width

The current study focused on participants with LTP, and the maxillary dental arch width at T1 before treatment of participants diagnosed with LTP in both the OMFT and no OMFT groups was approximately 33 mm. This was narrower than the 35.18 mm width in the control group in the present study and the 35.22 mm width in healthy children in a previous study (Table 1) [15]. However, both the OMFT and no OMFT groups had not only narrow dental arches, but also nasal airway obstruction (298.31 and 493.7 Pa). Hence, it was not possible to determine whether the LTP was due to narrow dental arches or nasal airway obstruction (Figure 2A,B, Table 1). However, the maxillary dental arch width did not differ according to posttreatment nasal airway obstruction and LTP (Figure 2C,D, Table 3). This suggests that nasal airway obstruction exerts a greater effect despite dental arch narrowing affecting LTP [14]. In addition, the maxillary dental arch width was not significantly different between the OMFT and no OMFT groups even after treatment, and both nasal obstruction and LTP were improved by OMFT. Collectively, these findings confirmed that LTP was not related to dental arch width and that nasal obstruction was a major factor in TLP. As such, we consider that improvement of nasal airway obstruction is essential for improving LTP. However, even if nasal obstruction improves, there might still be participants in whom LTP will not improve owing to habitual mouth breathing or the effect of the frenulum.

### 4.3 Nasal Mucosa Hypertrophy and Deviation of the Nasal Septum

This study focused on participants with LTP. The rate of improvement in nasal airway obstruction with RME alone was 48.4% (Table 2), lower than the 60%–70% rate reported in previous studies [27, 35, 36]. This difference may be due to the fact that the participants in the current study had more severe conditions than those in previous studies [27, 35, 36], as the current study involved children with LTP. Particularly, many participants (26 and 18 participants in the OMFT and no OMFT groups, respectively) had hypertrophic nasal mucosa and deviated nasal septum. However, there was no significant difference in the distribution of these participants between the OMFT and no OMFT groups, suggesting that the comparison results were not significantly affected by this factor. The current study examined the effects of OMFT in individuals with nasal mucosa hypertrophy (Table 2). Among the participants with nasal mucosa hypertrophy in the no OMFT group, only a few participants experienced LTP improvement. Meanwhile, the improvement rate for nasal airway obstruction was 25%, similar to the 31.6% rate for participants with nasal polyps in the no OMFT group reported by Sakoda et al. [16] However, the improvement rate in the OMFT group was high at 82.2%, clearly indicating that OMFT is particularly effective for improving nasal airway obstruction in patients with nasal mucosal hyperplasia. Furthermore, this study also examined the effects of OMFT in participants with deviated nasal septum (Table 2). The results showed that OMFT improved tongue posture, nasal mucosal hyperplasia and nasal airway obstruction, highlighting its possible application in patients with deviated nasal septum. Despite the small sample size, the findings underscore the effectiveness of OMFT in improving nasal airway obstruction and tongue posture in children.

### 4.4 Clinical Implications

The results of this study clarify the mechanisms through which OMFT effectively improves the outcomes of paediatric OSA posttreatment (e.g., airway expansion treatment such as adenotonsillectomy and RME), which have been previously unclear. Although a nasal airway is secured through adenotonsillectomy or RME, only the muscles for mouth breathing are developed, resulting in continued mouth breathing and no improvement in OSA. However, by strengthening the perioral muscles with OMFT, lip closure and nasal breathing become possible, the nasal airways are enlarged, nasal airway ventilation is improved and LTP is also improved. Therefore, acquiring muscle function for nasal breathing via OMFT after airway enlargement via RME or other modalities is important for the improvement of OSA.

### 4.5 Limitations

There are limitations to being able to accurately determine whether the tongue posture during imaging is the same as the resting posture and whether or not mouth breathing is present. However, we believe that the determination of the resting tongue posture was sufficiently reliable in the present study as participants were carefully selected based on the shape of the tongue dorsum and soft palate, the shape of the lips and jaw and other factors such as image blur. Research on the relationship of clinical findings of tongue posture and mouth breathing with CBCT data is needed. In the current study, OMFT was only applied as a standard modality by 2020. The dentition width diameter, tongue position, or nasal ventilation status at T1 was not significantly different between the OMFT and no OMFT groups, confirming that OMFT was randomly applied in the two groups.

### 4.6 Future Directions

The results of this study examined the effect of OMFT on tongue posture in participants who have underwent RME, which has been reported to effectively improve nasal airway obstruction [27]. Thus, the independent effect of OMFT is unclear. Future studies on the efficacy of OMFT in patients who have not underwent RME are necessary. This will also involve clarifying the mechanism of nasal airway obstruction improvement by OMFT (e.g., the cross‐sectional area of the nasal airway). Furthermore, the benefit of OMFT in improving tongue posture in children with OSA needs to be quantitatively examined.

## 5 Conclusion

OMFT improves LTP and nasal airway obstruction. The improvement of nasal airway obstruction by OMFT is highly likely to correlate with the improvement of LTP. Nasal airway pressure of ≥ 100 Pa (flow rate 200 mL/s, nasal airway resistance 0.5 Pa/mL/s) affects LTP.

## Author Contributions

K.M. contributed to conception and design, data acquisition and interpretation, drafted and critically revised the manuscript. T.K. contributed to funding aquisition and data analysis, W.K. and Y.S. contributed to data aquisition and analysis, Y.Y. and K.U. contributed to data acquisition. H.I. and R.K. contributed to data aquisition and conception and design, critically revised the manuscript. T.I. contributed to conception and design, data acquisition and interpretation, critically revised the manuscript. All authors have their final approval and agree to be accountable for all aspects of the work.

## Ethics Statement

This study was approved by the Institutional Review Board of Tokushima University Hospital (4393‐4), following the Declaration of Helsinki.

## Conflicts of Interest

The authors declare no conflicts of interest.

## Data Availability

The data that support the findings of this study are available from the corresponding author upon reasonable request.
